# Prodomain-driven enzyme dimerization: a pH-dependent autoinhibition mechanism that controls *Plasmodium* Sub1 activity before merozoite egress

**DOI:** 10.1128/mbio.00198-24

**Published:** 2024-02-22

**Authors:** Mariano Martinez, Anthony Bouillon, Sébastien Brûlé, Bertrand Raynal, Ahmed Haouz, Pedro M. Alzari, Jean-Christophe Barale

**Affiliations:** 1 Unité de Microbiologie Structurale, Institut Pasteur, CNRS UMR 3528, Université Paris Cité, Paris, France; 2 Plate-forme de Biophysique Moleculaire-C2RT, Institut Pasteur, CNRS UMR 3528, Université Paris Cité, Paris, France; 3 Plate-forme de Cristallographie-C2RT, Institut Pasteur, CNRS UMR 3528, Université Paris Cité, Paris, France; NIAID/NIH, North Bethesda, Maryland, USA

**Keywords:** malaria, *Plasmodium*, subtilin-like protease, pro-domain, enzyme regulation, 3D structure, egress

## Abstract

Malaria symptoms are associated with the asexual multiplication of *Plasmodium falciparum* within human red blood cells (RBCs) and fever peaks coincide with the egress of daughter merozoites following the rupture of the parasitophorous vacuole (PV) and the RBC membranes. Over the last two decades, it has emerged that the release of competent merozoites is tightly regulated by a complex cascade of events, including the unusual multi-step activation mechanism of the pivotal subtilisin-like protease 1 (Sub1) that takes place in three different cellular compartments and remains poorly understood. Following an initial auto-maturation in the endoplasmic reticulum (ER) between its pro- and catalytic domains, the Sub1 prodomain (PD) undergoes further cleavages by the parasite aspartic protease plasmepsin X (PmX) within acidic secretory organelles that ultimately lead to full Sub1 activation upon discharge into the PV. Here, we report the crystal structure of full-length *P. falciparum* Sub1 (PfS1_FL_) and demonstrate, through structural, biochemical, and biophysical studies, that the atypical *Plasmodium-*specific Sub1 PD directly promotes the assembly of inactive enzyme homodimers at acidic pH, whereas Sub1 is primarily monomeric at neutral pH. Our results shed new light into the finely tuned Sub1 spatiotemporal activation during secretion, explaining how PmX processing and full activation of Sub1 can occur in different cellular compartments, and uncover a robust mechanism of pH-dependent subtilisin autoinhibition that plays a key role in *P. falciparum* merozoites egress from infected host cells.

**IMPORTANCE:**

Malaria fever spikes are due to the rupture of infected erythrocytes, allowing the egress of *Plasmodium* sp. merozoites and further parasite propagation. This fleeting tightly regulated event involves a cascade of enzymes, culminating with the complex activation of the subtilisin-like protease 1, Sub1. Differently than other subtilisins, Sub1 activation strictly depends upon the processing by a parasite aspartic protease within acidic merozoite secretory organelles. However, Sub1 biological activity is required in the pH neutral parasitophorous vacuole, to prime effectors involved in the rupture of the vacuole and erythrocytic membranes. Here, we show that the unusual, parasite-specific Sub1 prodomain is directly responsible for its acidic-dependent dimerization and autoinhibition, required for protein secretion, before its full activation at neutral pH in a monomeric form. pH-dependent Sub1 dimerization defines a novel, essential regulatory element involved in the finely tuned spatiotemporal activation of the egress of competent *Plasmodium* merozoites.

## INTRODUCTION

Malaria is the most devastating parasitic disease in the world, and its control is compromised by the constant selection of multi-resistances to existing treatments (1). Transmitted through the bite of the *Anopheles* mosquito vector, the parasites quickly reach the liver, where they invade host hepatocytes in which they asexually multiply in merozoites within a parasitophorous vacuole (PV). Following this asymptomatic phase, merozoites egress from host hepatocytes, a process in which the *Plasmodium* subtilisin-like Sub1 protease plays a key role (2, 3), to reach the blood and invade host red blood cells (RBCs) to initiate the erythrocytic cycle responsible for the symptoms of malaria. As in the hepatocytes, the parasites grow and asexually multiply by schizogony within a parasitophorous vacuole in the erythrocyte cytoplasm in 24–72 h, depending on *Plasmodium* species. The newly formed merozoites egress after the rupture of the PV and erythrocytic membranes to invade new RBCs. The egress of merozoites is a finely regulated and multi-step event initiated by a kinase-mediated (4, 5) intracellular shift of calcium that promotes the discharge of Sub1 from a merozoite secretory organelle, the exoneme, to the PV (6). Upon activation, Sub1 participates in PV membrane disruption, activates soluble proteases of the SERA family involved in the rupture of the erythrocytic membrane, and matures different merozoite surface proteins important for RBC invasion (7–9). Despite the central role of Sub1 in the cascade of enzymes that controls the egress of mature merozoites from host hepatocytes and erythrocytes (10, 11), important mechanistic questions on the protease transport and activation remain unknown.

Sub1 belongs to the large subtilisin, or subtilase, family of serine proteases that are involved in a broad spectrum of biological functions (12–14). Subtilisins constitute the S8 family in clan SB of serine proteases (15), which is divided into the S8A and S8B sub-families corresponding respectively to prokaryotic enzymes (12, 13), known to display a broad specificity, and to eukaryotic kexin-like enzymes (14). Among the latter, the Ca^2+^-dependent prohormone convertases (PCs) precisely mature polypeptidic precursors after mono or di-basic residues, producing functional hormones, also activating bacterial toxins or surface proteins of various viruses (16–18). As with most other proteases, subtilisins are synthesized as a zymogen with a core structure composed of at least two domains, the catalytic domain, and a dual function prodomain. The catalytic domain is organized around a catalytic His-Asp-Ser triad, with a fourth conserved Asn that is part of the oxy-anion hole that stabilizes the negative charge of the tetrahedral intermediate formed between the enzyme and its substrate (19). Subtilisin prodomains act both as intramolecular chaperones for and potent inhibitors of their cognate catalytic domains (20–23). In both bacteria and eukaryotic cells, primary automaturation occurs between the subtilase catalytic domain and its prodomain during their secretion, when exposed to variations of pH and/or Ca^2+^-concentration that induce complex disassembly and subsequent degradation of the prodomain by the active enzyme (23, 24).

Subtilisins are highly regulated to ensure that enzyme activation is triggered at the right place and time. In bacteria, the removal of subtilisin prodomains usually depends upon two distinct autoproteolytic cleavages, each with a different pH optimum (25, 26). For eukaryotic PCs, specific histidines located in prodomains have been shown to act as pH sensors to trigger subtle conformational changes in the desired cellular compartment to release and degrade their prodomain. Furin His_69_ and PC1/3 His_72_/His_75_ have been shown to play this role during their secretion in the mildly acidic pH of the trans-Golgi network (pH ≈ 6.5) and in more acidic mature secretory granules (pH ≈ 5.5), respectively (27, 28). While pH also plays an important role to remove the prodomain of plant subtilisins (29), these enzymes also frequently contain, between the histidine and the serine of their catalytic triad, a protease-associated (PA) domain described to trigger protein-protein interactions (30). While involved in extended substrate recognition (31), the PA domain also regulates the activity of the SlSBT3 plant subtilisin, promoting its vital homodimerization to maintain the active site accessible to the substrate (32, 33). Finally, subtilisins may also be regulated in *trans*, by prodomain-like polypeptides that inhibit cellular enzymes found in yeast (34), plant (35), or in the apicomplexa human-infecting parasites *Toxoplasma gondii* (36, 37) or *Plasmodium falciparum* (38).

*Plasmodium* sp. Sub1 subtilases are synthesized as inactive precursors of ≈80 kDa in which a bacterial-like catalytic domain (39, 40) is preceded by an atypical prodomain (PD) composed of two regions: a parasite-specific N-terminal region of unknown function followed by a C-terminal region structurally and functionally similar to canonical bacterial subtilisin PDs. Upon translocation into the endoplasmic reticulum (ER), Sub1 undergoes autocatalytic cleavage to yield a C-terminal 54 kDa intermediate (p54) that remains noncovalently bound to the cleaved PD of 31 kDa (p31) (41). During exoneme transport to the PV, Sub1 undergoes further processing by the Asp protease plasmepsin X (PmX) (42–44), which has recently been shown to directly cleave both the Sub1 PD and the catalytic domain (45). However, it remains unclear why PD processing within the exoneme is spatially dissociated from full Sub1 activation, which only occurs at a later stage, upon discharge into the PV. Here, we report the crystal structure of full-length *P. falciparum* Sub1 (PfS1_FL_) and demonstrate, through comparative structural analysis substantiated by biochemical and biophysical studies, that the atypical Sub1 PD is directly responsible for the assembly of inactive enzyme homodimers at acidic pH. Our results uncover a novel robust mechanism of pH-dependent subtilisin autoinhibition, which plays a crucial role in the tight regulation of *Plasmodium* merozoite egress from host cells and accounts for the different compartmentalization of PmX processing and full Sub1 activation.

## RESULTS

### The overall structure of full-length Sub1

**Fig 1:**
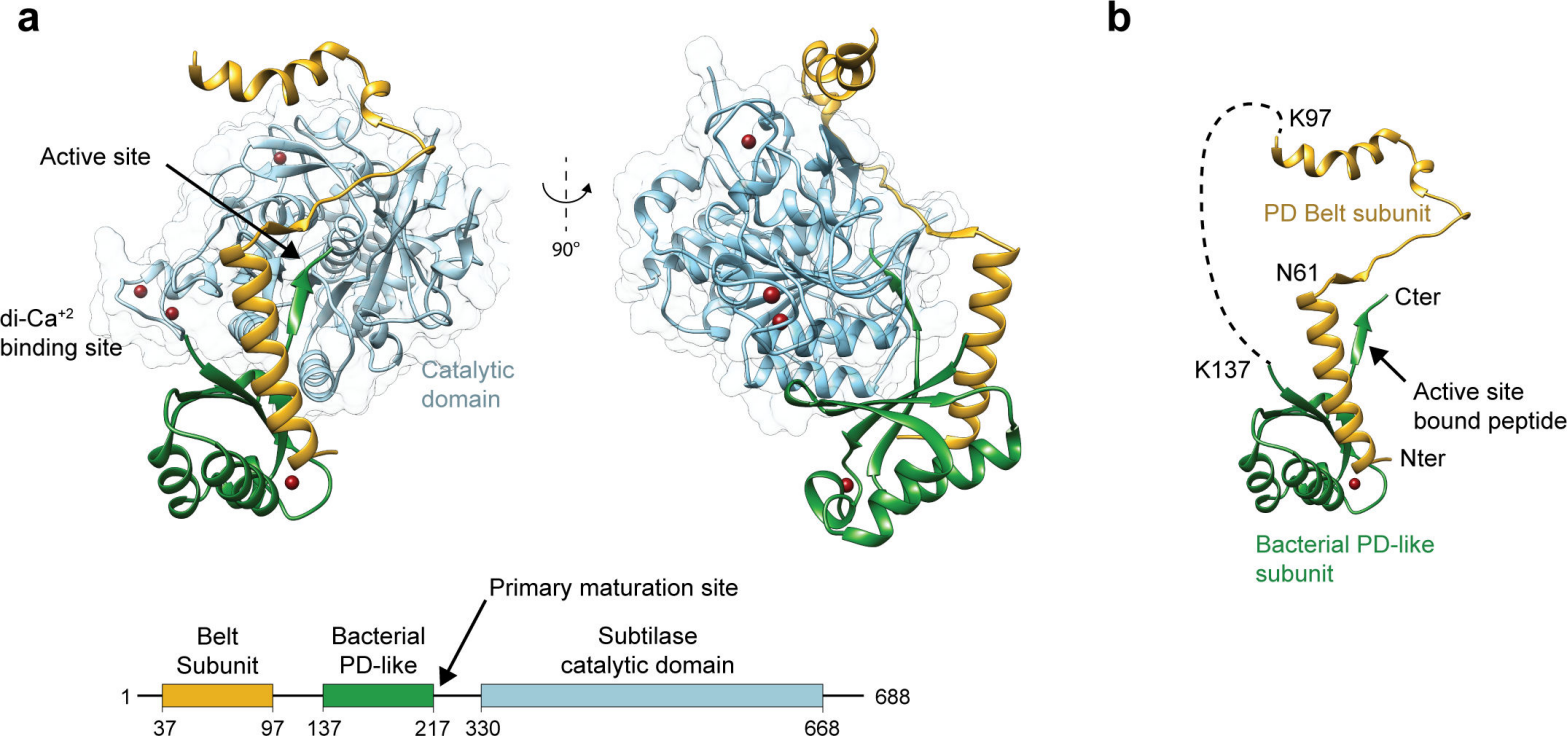
Overall structure of full-length *P. falciparum* Sub1 (PfS1_FL_). (a) The catalytic domain (in cyan) is tightly bound to its cognate PD made up of two distinct subunits, the N-terminal belt subunit (yellow) and the bacterial PD-like subunit (green). The PD connecting segment between the two subunits (residues 116–153) and the N-terminal region of the mature catalytic domain (residues 236–347) are not visible in the electron density and are presumably disordered in the crystals. The schematic domain organization of PfS1_FL_, with the three structured domains, is shown below the structure. (b) Detailed structure of the Sub1 PD color-coded as in panel (a). The active site-bound propeptide and specific residues discussed in the text are labeled.

The X-ray structure of recombinant full-length protease Sub1 from *P. falciparum* (PfS1_FL_) expressed in insect cells was determined by molecular replacement methods at 3.09 Å resolution (Table 1). As expected, the structure revealed a subtilisin-like catalytic core (residues 330–668) tightly bound to its cognate PD composed of two distinct subunits (residues 37–97 and 137–217, Fig. 1a). Complex formation between the catalytic core and the PD buries ~2,350 Å^2^ of surface area from each moiety, which roughly duplicates the values observed for the similar interface in bacterial subtilisin (46). The overall architecture is very similar to that of the full-length *Plasmodium vivax* Sub1 [PvS1_FL_ (47), PDB code 4tr2] (Fig. S1). The two structures can be superimposed with an overall rmsd of 0.78 Å for 462 equivalent Cα atomic positions. All structural features characteristic of *Plasmodium* subtilases are conserved, including the three high-affinity calcium-binding sites in the catalytic domain, two of which are specific to *Plasmodium* subtilases, and a fourth calcium-binding site in the PD, all of which were previously described for PvS1_FL_ (47). Another common feature is that the PD had undergone autocleavage at its primary maturation site (Asp 217 in PfS1_FL_ and Asp202 in PvS1_FL_) but remained tightly associated to the catalytic core in the crystal, partially occupying the active site.

**TABLE 1:** Crystallographic data

Data collection	
Space group	*P*2_1_2_1_2_1_
Cell dimensions	
*a*, *b*, *c* (Å)	86.58, 107.16, 137.68
Resolution (Å)^*a*^	43–3.09 (3.30–3.09)
Number of unique reflections	24,055 (4,147)
*R*_merge_	0.104 (0.544)
*R*_pim_	0.060 (0.317)
*I*/*σ* (*I*)	11.1 (2.4)
Completeness (%)	99.2 (96.2)
Redundancy	3.9 (3.9)

Refinement	
Resolution (Å)	3.09
*R*_work_/*R*_free_	0.170/0.228
No. of atoms	
Protein	7,523
Ligands	33
Temperature factors (Å^2^)	
Protein	77.3
Ligands	107.1
Root mean square deviations	
Bond lengths (Å)	0.004
Bond angles (°)	0.70
Ramachandran outliers (%)	0
Rotamer outliers (%)	0.35

^*a*^ Values in parenthesis refer to the highest recorded resolution shell.

The Sub1 PD is made up of two distinct subunits and displays an atypical folding (Fig. 1b). The structural core consists of an N-terminal α-helix that interacts through an interface of 700 Å^2^ with an α/β subunit similar to bacterial subtilisin PDs (46), which includes the C-terminal peptide extension that remains bound to the protease active site after primary maturation. The N-terminal helix is connected to the first β-strand by a 75 residues-long loop that is largely devoid of secondary structure and mostly disordered, except for its first part that embraces the catalytic domain as a belt (Fig. 1a). The structural subunit comprising the N-terminal helix and the ordered part of the adjacent loop (residues 37–97, referred to here as the belt subunit) is also present in PvS1_FL_ and is highly conserved in all *Plasmodium* species (47), suggesting that it fulfills an important, as yet unknown, functional role. This subunit was missing in the previously reported structure of *P. falciparum* Sub1 in complex with a specific antibody (PDB code 4lvn) (48), possibly due to N-terminal truncation of the protein following chymotrypsin treatment during purification. On the other hand, the catalytic domains from both *P. falciparum* Sub1 structures (PfS1_FL_, 4lvn) are very similar to each other, with an rmsd of 0.43 Å for 329 equivalent Cα positions.

### The Sub1 prodomain mediates pH-dependent protein dimerization

**Fig 2:**
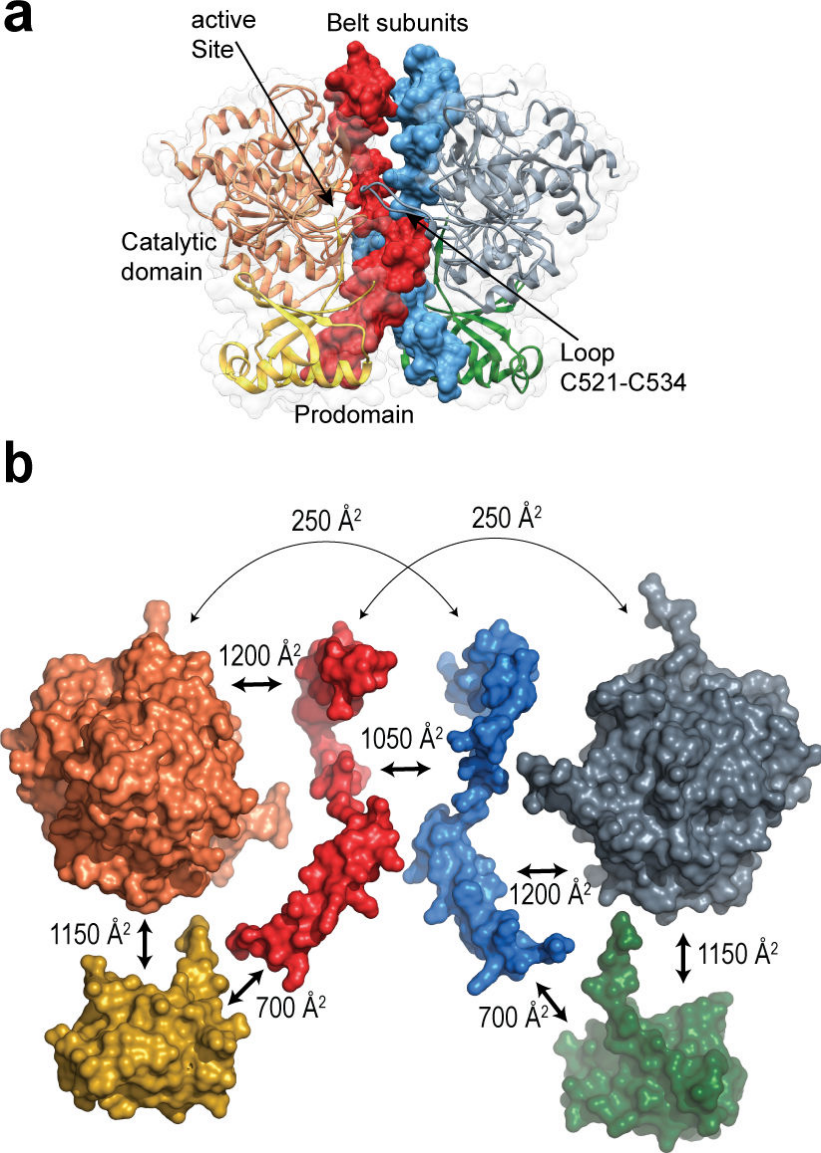
The *Plasmodium*-specific belt subunit mediates Sub1 dimerization. (a) Crystallographic dimer of PfS1_FL_ showing the interactions between the two protomers. The belt subunits (shown in molecular surface representation in red and blue) occupy the center of the dimer interface. (b) Exploded view of the PfS1_FL_ homodimer showing the interface contact surfaces between the different subdomains. The catalytic domains are shown in orange and gray, the bacterial PD-like subunits in yellow and green, and the belt subunits in red and blue. Due to the presence of the belt subunit, the total contact surface between the PD and the catalytic domain within a single Sub1 monomer (>3,000 Å^2^) roughly duplicates the interface observed in other subtilisins.

A large majority of subtilisin-like proteases are active as monomers (49). However, PfS1_FL_ crystallized as a homodimer at acidic pH, with an interfacial area of ~1,600 Å^2^ that involves extensive intermolecular hydrophobic and H-bond interactions. The PD is largely responsible for fastening together the Sub1 homodimer, as the belt subunits from both protomers occupy the center of the dimer, sandwiched between the two catalytic domains with their active sites facing each other (Fig. 2a). Intermolecular interactions between the belt subunits from the two protomers (through an interface of 1,050 Å^2^) account for roughly two-thirds of the total dimer interface (Fig. 2b). Other intermolecular contacts involve the interactions between the catalytic domain from one protomer with the belt subunit from the opposite protomer (250 Å^2^ interface). In contrast, the two catalytic domains are not directly in contact with each other, except for the tip of the prominent loop C521-C534, which protrudes out from one monomer to clamp the opposite catalytic domain (Fig. 2a). This loop, which is conserved in *Plasmodium* Sub1 orthologs but absent from other subtilisins, is stabilized by a disulfide bridge (Cys521-Cys534) at its basis and is adjacent to the oxyanion hole residue Asn520. Previous work had suggested that this redox-sensitive disulfide could act as a regulator of protease activity in the parasite (48).

**Fig 3:**
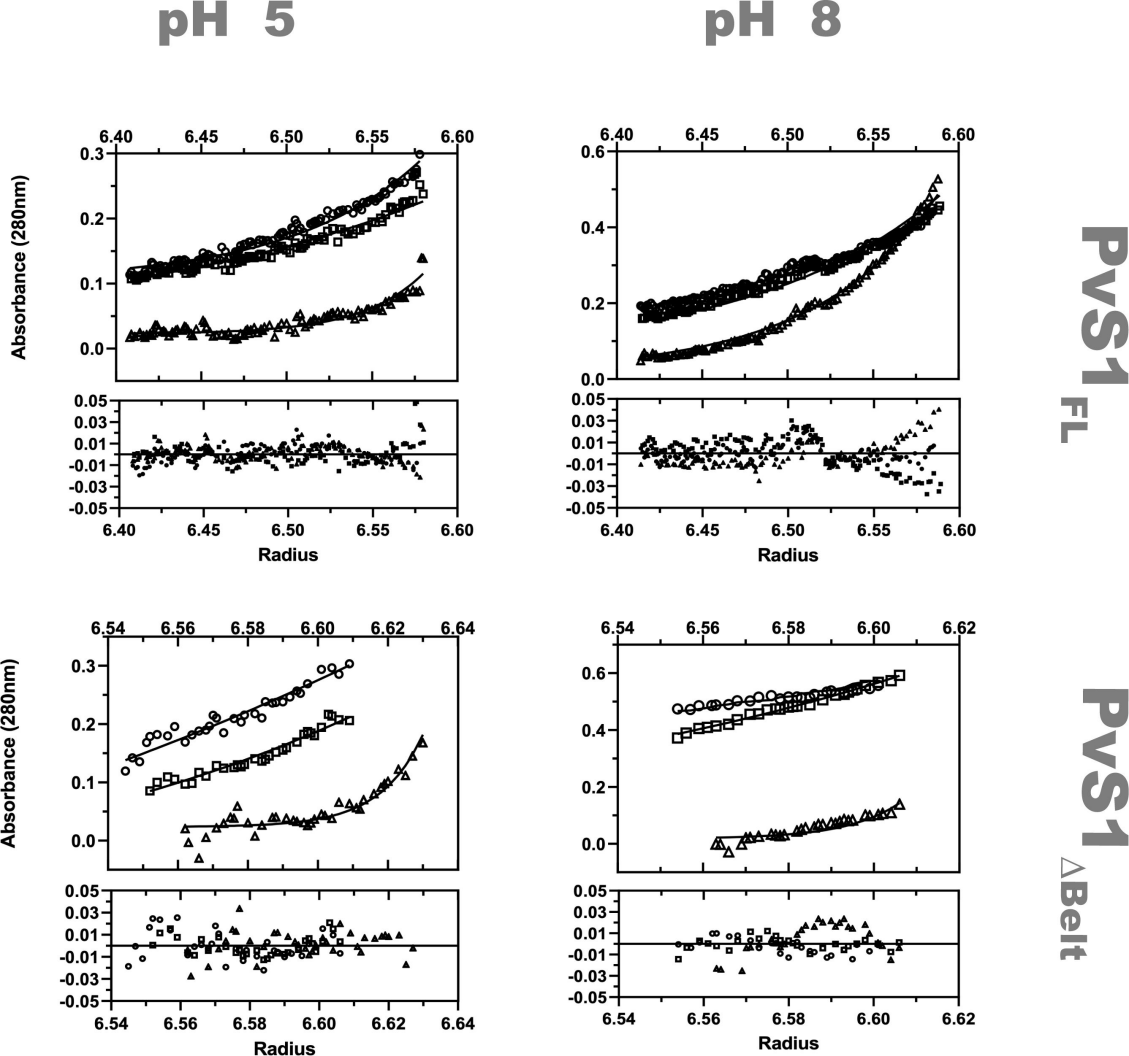
pH-dependent dimerization of Sub1. Analytical ultracentrifugation (AUC) sedimentation equilibrium analysis on purified PvS1_FL_ (top panels) and PvS1_ΔBelt_ (bottom panels) at pH 5 (left panels) and pH 8 (right panels). AUC profiles were recorded at 20°C using different concentrations at three rotor speeds 9,000 rpm (squares), 13,000 rpm (circles), and 16,000 rpm (triangles). For clarity, only velocity traces of proteins at 5 µM are shown. For every experiment, the upper panel shows the sedimentation equilibrium profiles with the lines of best fit obtained for a single species model (molecular weights are shown in Table 2), and the lower panel shows the data fitting residuals.

Interestingly, a similar face-to-face dimer (rmsd of 1.58 Å for 920 equivalent residues) had been observed in crystals of PvS1_FL_ (Fig. S2a), which were also grown in acidic conditions (pH 4.2–5) but belong to a different crystal form (47). In PvS1_FL_, however, a different crystal lattice interface (Fig. S2b) presented a larger contact surface area (~1,800 Å^2^ compared to ~1,400 Å^2^ for the face-to-face dimer). To experimentally investigate whether any of these interactions could stabilize an oligomeric form of Sub1 in solution, we carried out analytical ultracentrifugation (AUC) studies of recombinant PvS1_FL_. The protein was found to form a stable homodimer in solution at pH 5 but behaves as a monomer at pH 8 under otherwise identical conditions (Fig. 3; Table 2). These results imply that (i) Sub1 dimerization is pH-dependent, as an increase of pH promoted dimer dissociation, and (ii) one of the two crystallographic dimers observed for PvS1_FL_ (Fig. S2) must be stable in solution. To elucidate which one, we repeated the AUC experiment on an N-terminal truncated form of PvS1 lacking the belt subunit (PvS1_ΔBelt_), as this subunit is largely involved in the face-to-face dimeric interface but not in the back-to-back dimer (Fig. S2). Deletion of the N-terminus rendered the protein monomeric in solution at acidic pH (Fig. 3; Table 2), indicating that the face-to-face dimer observed for both the PvS1_FL_ and PfS1_FL_ structures in completely different crystal environments does correspond to a stable Sub1 dimer in solution.

**TABLE 2:** pH-dependent oligomerization of PvS1_FL_ and PvS1_ΔBelt_*^a^*

Protein	Theor. MW (monomer)	pH 5	pH 8
PvS1_FL_	67.71 kDa	135.8 ± 2.8 kDa	67.9 ± 1.1 kDa
PvS1_ΔBelt_	56.57 kDa	57.5 ± 0.2 kDa	57.5 ± 4.5 kDa

^*a*^ Molecular weight determination of PvS1_FL_ and PvS1_ΔBelt_ variants by analytical ultracentrifugation sedimentation equilibrium at pH 5 and pH 8, corresponding to the AUC profiles shown in Fig. 3. The theoretical molecular weights were determined from the amino acid sequence of the proteins. Error estimates were calculated using a Monte-Carlo simulation of 10,000 replicates.

**Fig 4:**
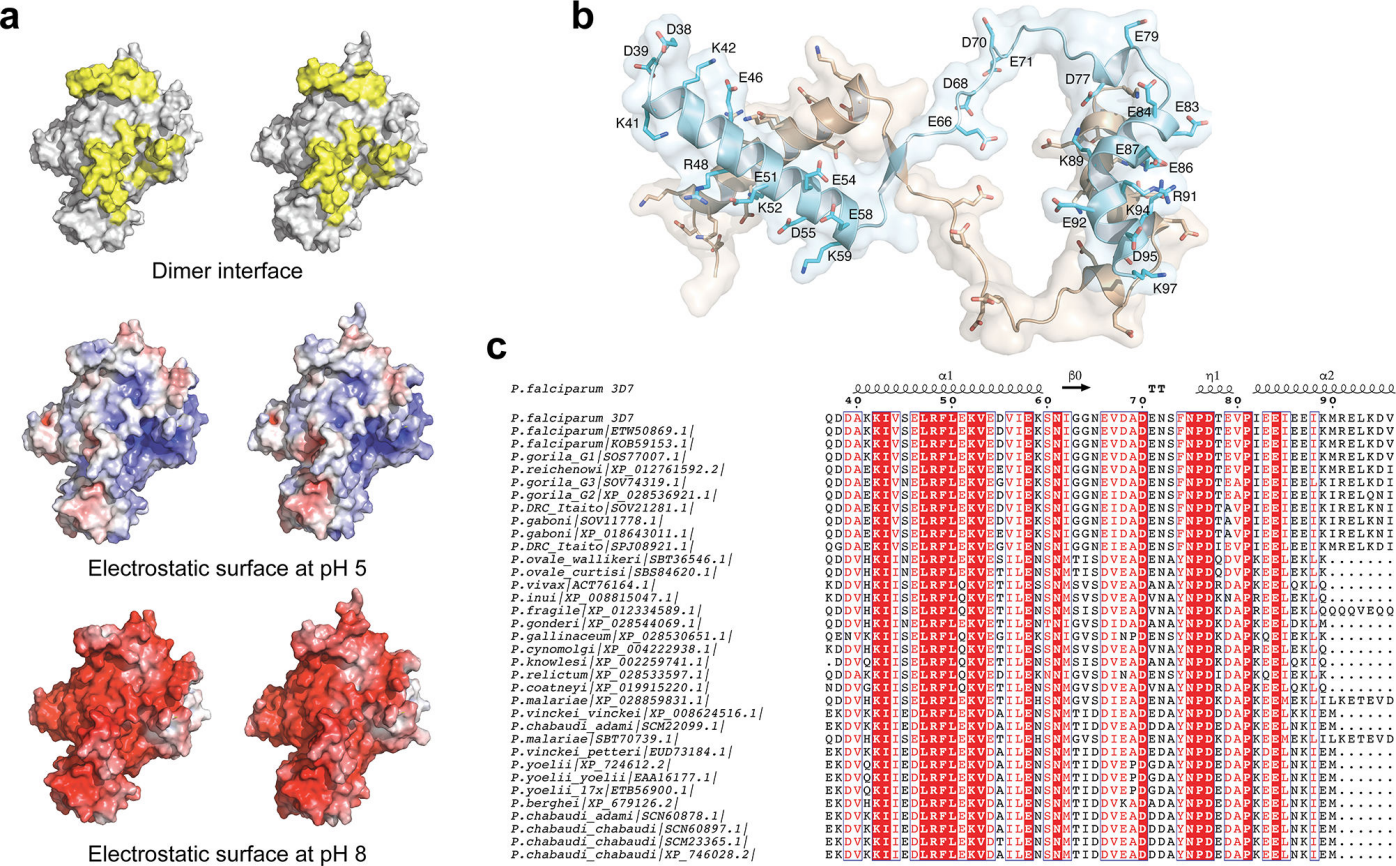
pH-dependent interactions. (a) Open book view of the dimer in surface representation (top) with the interface residues colored yellow. Below, gradient visualization from red (−10 kT/e) to blue (+10 kT/e) of the electrostatic surface at pH 5 and 8, calculated with the APBS server (52) based on residue pKa determined by PROPKA (51). (b) Cartoon representation of the belt subunits from both protomers, which occupy the center of the homodimer. In this orientation, the catalytic domains (omitted from the figure) are respectively below and above the plane of the paper. All charged residues (Asp, Glu, Arg, and Lys) are shown in stick representation and labeled for one of the two subunits. (c) Conserved amino acid sequence of the belt subunit from different *Plasmodium* Sub1 orthologs.

At neutral pH, the entire dimerization interface is predicted to be negatively charged (Fig. 4a), which likely results in Coulombic repulsion and prevents dimerization. Largely buried at the center of the interface, the belt subunit contains an important concentration of negatively charged residues (Fig. 4b). In PfS1_FL_, 18 out of 61 ordered residues in this region (29.5%) are Asp or Glu, whereas in the rest of the structure, this percentage is significantly lower, 12.4%, matching the average amino acid protein composition in whole proteomes (50). Importantly, most of these negatively charged belt positions are largely or strictly conserved in *Plasmodium* Sub1 orthologs (Fig. 4c), and some of them are partially occluded from the solvent upon dimerization. At pH 5 the dimerization interface of PfS1 is predicted to become neutral (Fig. 4a), thus favoring dimerization. Several negatively charged residues that belong to or interact with the belt subunit are predicted to become protonated at pH 5 using the PROPKA program (51). For example, a cluster of seven glutamic acids (E38, E46, E51, E187, E202, E209, and E495), five of which would be protonated at pH 5, participate in the interaction of the N-terminal belt helix with the bacterial-like pro-region and the catalytic domain from the same molecule (Fig. S3). The close proximity between their carboxylate groups suggests that, at neutral pH, Coulombic repulsion would destabilize this association. Moreover, eight histidines are predicted to change from neutral to positively charged at pH 5, three of which (H464, H523, and H596) are part of the homodimer interface. These pH-induced charge transitions might allow the formation of intermolecular salt bridges at acidic pH, as, for example, H464 and H596 from each protomer respectively face D68 and E71 from the other protomer in the dimer. In summary, these changes in surface charge can explain the observed Sub1 transition in solution from a single dimeric species at acidic pH to a single monomeric form at neutral pH.

### Protein dimerization as a robust mechanism for enzyme autoinhibition

**Fig 5:**
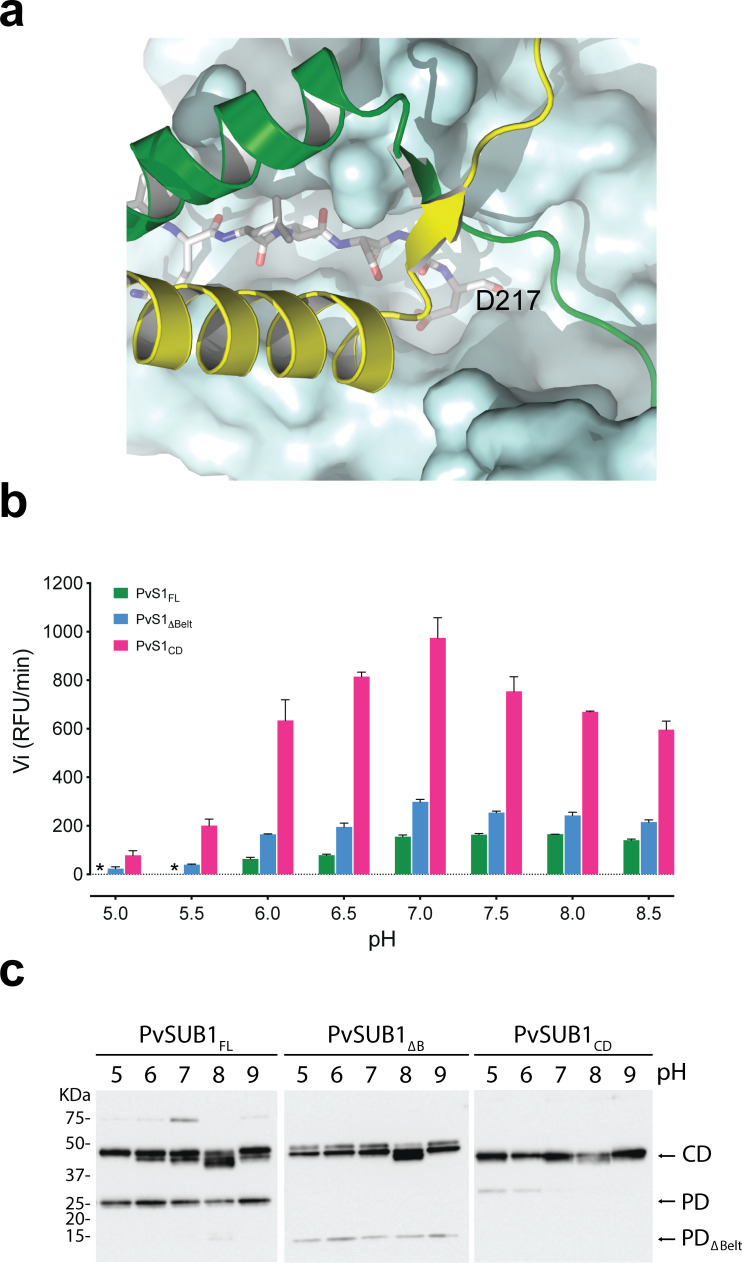
Protein dimerization as a robust inhibition mechanism. (a) The active site cleft of the catalytic domain (represented as a blue molecular surface) containing the cleaved prodomain peptide (in stick representation) is completely blocked by the two belt subunits (yellow and green) in the homodimer. The second catalytic domain (above the plane of the paper in this view) has been omitted for clarity. (b) pH dependence of enzyme activity for different PvS1 constructs as indicated. The initial hydrolysis rates (arbitrary units) are shown for the actual final pH of the assay buffer at 37°C, determined as described under “Experimental Procedures.” In the plots, each data point represents the mean value of three independent assays and an asterisk (*) indicates undetectable activity. (c) SDS-PAGE for the three tested constructs at different pH values. The bands corresponding to the catalytic domain (CD), the full prodomain (PD), and the prodomain lacking the belt subunit (PD_ΔBelt_) are indicated on the right.

PD-mediated protein dimerization traps the cleaved propeptide within the active site and completely blocks the access to (or release from) the substrate-binding clefts from both protomers (Fig. 5a), indicating that homodimerization could serve as an efficient mechanism for Sub1 autoinhibition. Enzymatic assays at different pH values tend to confirm this hypothesis. Recombinant PfS1 was reported to be active in a wide range of pH values, with optimal activity at around pH 8 (53), and we obtained similar results for PvS1. To investigate the inhibitory effects of the PD structural subunits on PvS1 activity at different pH values, we carried out enzyme assays on the full-length enzyme (PvS1_FL_), a construct lacking the belt subunit but containing the bacterial PD-like subunit (PvS1_ΔBelt_) and the catalytic domain alone (PvS1_CD_). For all these constructs, the enzymatic activity could be detected in a wide range of tested pH values, with a peak at around pH 7 (Fig. 5b). As expected, PvS1_CD_, which corresponds to the fully active protease, has a significantly higher activity than the two other constructs, which are partially or totally inhibited by the bound PD and whose activity can still be measured possibly because a fraction of the processed catalytic domains have lost their bound cognate PD during purification (53). Interestingly, no detectable activity was observed for dimeric PvS1_FL_ at the most acidic conditions (pH 5–5.5), whereas a small but significant activity could still be measured at the same pH for both PvS1_ΔBelt_ and PvS1_CD_ (both unable to dimerize), highlighting the robustness of pH-induced dimerization as an autoinhibition mechanism. As a control, mobility in SDS-PAGE of the three constructs used above was assayed at different pH values (Fig. 5c). Consistent with the activity measurements (Fig. 5b), the catalytic domain of PvS1_FL_ migrates as a single band only at the most acidic pH, where the protease has no detectable activity, but migrates as a double band in all other cases where a residual activity could be detected.

### Sub1 detection in *P. falciparum* merozoites prior to egress from infected erythrocytes

**Fig 6:**
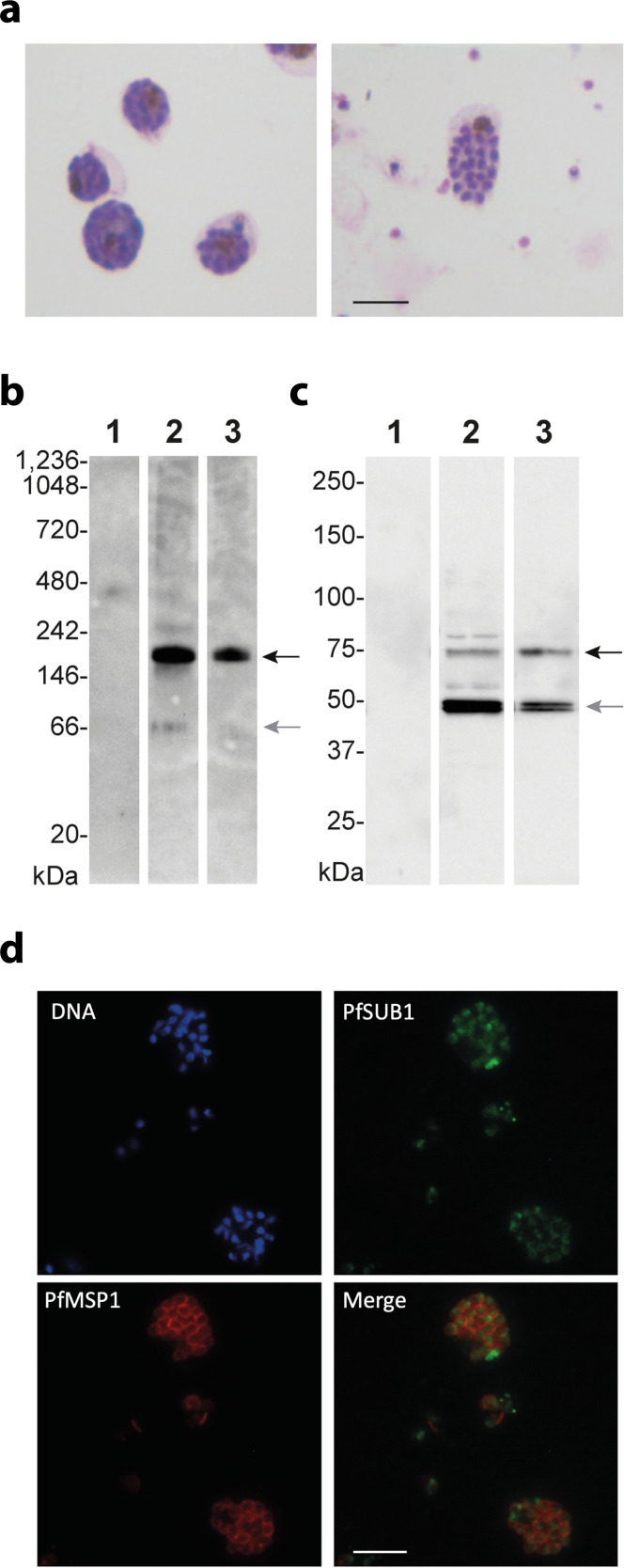
A dimeric form of PfS1 in *P. falciparum* schizonts prior to the merozoites egress. (a) Giemsa-stained parasite cultures corresponding to mature *P. falciparum* schizonts (left panel) and 5 h later containing mature schizonts and free merozoites (rigth panel) that have egressed from infected erythrocytes. (b) Western blot of native protein extracts prepared from uninfected RBCs (lane 1), synchronous culture of mature *P. falciparum* schizonts prior to the egress of merozoites (lane 2, corresponding to the left panel in Fig. 6a) and a mixture of mature schizonts and free merozoites (lane 3, corresponding to the right panel in Fig. 6a) revealed using anti-PfS1 antibodies. The multimeric form compatible with a PfS1 dimer and the form compatible with the PfS1 catalytic domain are indicated by black and gray arrows, respectively. Native protein molecular weights (MW) are indicated in kDa. (c) Western blot of protein extracts migrated on denaturating SDS-PAGE prepared from uninfected human RBCs (lane 1), synchronous culture of mature *P. falciparum* schizonts prior to the egress of merozoites (lane 2, corresponding to the left panel in Fig. 6a) and a mixture of schizonts and free merozoites (lane 3, corresponding to the right panel in Fig. 6a) revealed using anti-PfS1 antibodies. Traces of the precursor of full-length PfS1 and the most abundant maturated forms corresponding to the PfS1 catalytic domain are indicated by black and gray arrows, respectively. Protein molecular weights (MW) are indicated in kDa. (d) Immunofluorescence assays using DNA-labeling of the nuclei of differentiated intra-schizonts merozoites (Hoescht, blue), anti-PfS1 antibodies (green), and antibodies raised against the Merozoite Surface Protein 1 (MSP 1, red). The bottom right panel shows an overlay of PfS1 and PfMSP1 labeling. The scale bar represents 5 µm. The results shown in this figure are representative of several independent experiments.

To gain some insights into the behavior of native Sub1 within the parasites, proteins have been prepared from a culture of *P. falciparum* enriched in mature segmented schizonts containing merozoites prior to their egress from infected RBCs (Fig. 6a, left panel) or from a culture at the time of merozoite egress, composed of both mature schizonts and free merozoites (Fig. 6a, right panel). Following the migration of proteins on polyacrylamide gels under native conditions and transfer on nitrocellulose, antibodies raised against PfS1 revealed a main form with apparent molecular weights of approximately 180 kDa (Fig. 6b, lanes 2 and 3) that corresponds to the dimeric form of full-length PfS1 (Mw 77 kDa). A fainter signal at 66 kDa, which could correspond to PD-free catalytic domain of PfS1 (Mw 53 kDa), is detected only in schizonts prior to merozoite egress under native conditions (Fig. 6b, lane 2). Western blot analysis of the same protein extracts separated by denaturating SDS-PAGE detected the expected full-length precursor and the free catalytic domain in both parasite cultures (Fig. 6c, lanes 2 and 3), although the signal corresponding to PfS1 catalytic forms appeared fainter in the culture containing merozoites that have already egressed from infected RBCs (Fig. 6c, lane 3). As shown by immunofluorescence analysis, the signal revealed by anti-PfS1 antibodies shows a punctated labeling (Fig. 6d), consistent with a location within the merozoite secretory vesicles (exoneme). Taken together, these observations suggest that, prior to activation, Sub1 exists primarily as a dimer in the parasite.

## DISCUSSION

**Fig 7:**
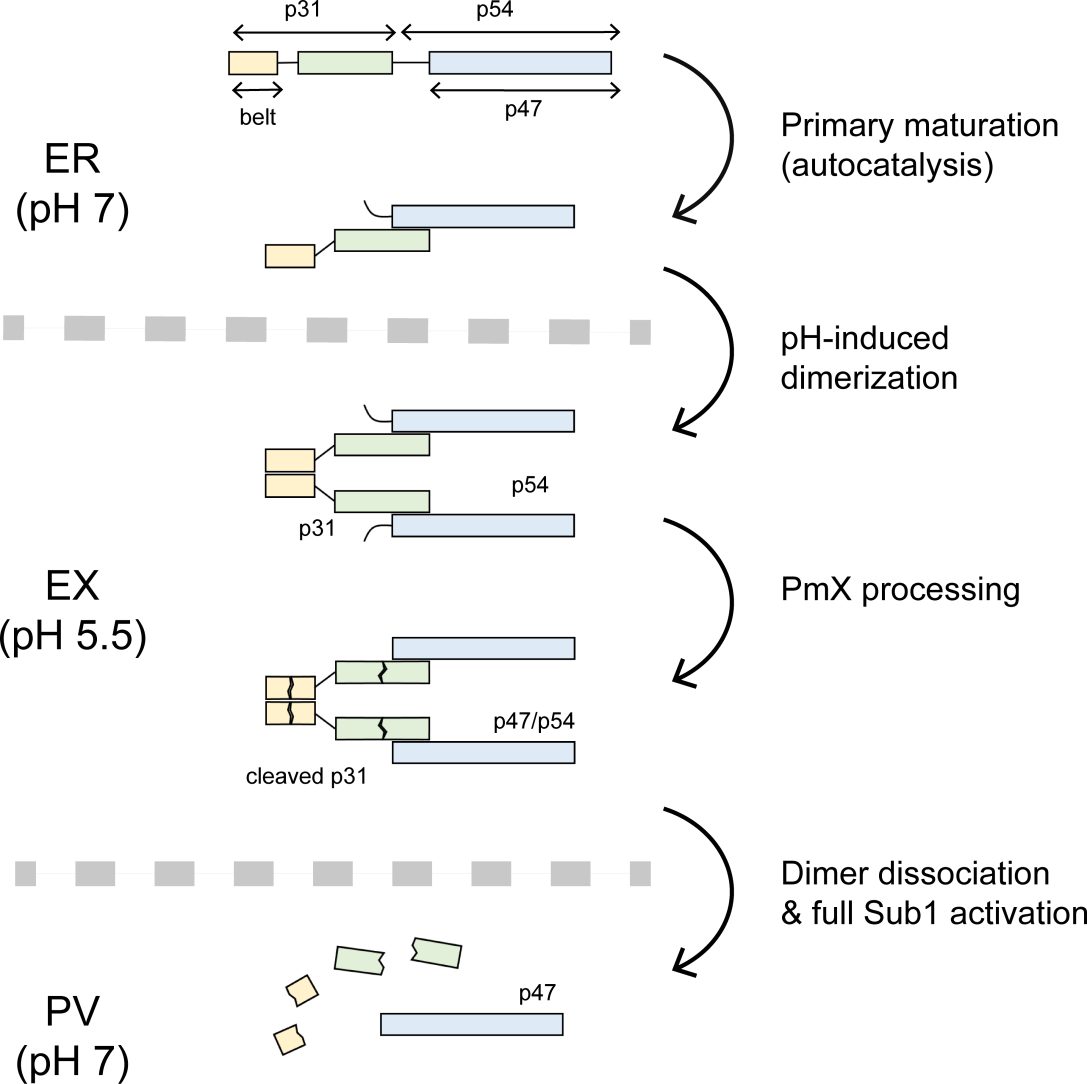
Schematic representation of the proposed activation model for *P. falciparum* Sub1. Primary maturation of Sub1 into the PD (p31) and the catalytic domain (p54) species takes place early in the endoplasmic reticulum (ER). The acidic environment of the exoneme (EX) induces the formation of the PD-mediated autoinhibited Sub1 dimer, which would remain inactive upon cleavage by plasmepsin X (PmX). Upon discharge into the rather neutral parasitophorous vacuole (PV), the higher pH dissociates the dimer leading to full Sub1 activation. The conversion of the catalytic domain from the p54 to the p47 species can be obtained either by direct PmX cleavage in the exoneme or by autocatalysis in the PV.

Like other subtilisins, Sub1 is synthesized as an inactive precursor made up of a PD bound to the catalytic domain. Unlike other subtilisins, however, Sub1 is characterized by an atypical trifunctional PD capable not only of facilitating protein folding and inhibiting the protease (54), but also of inducing pH-dependent protein dimerization, as we have shown here. Primarily mediated by the belt subunit, this later function ensures a robust inhibition mechanism during Sub1 transport in the acidic environment of the exoneme, and possibly explains the essentiality of the *Plasmodium*-specific N-terminal belt extension for the growth of *P. berghei* erythrocytic stages *in vivo* (47). Taken together with previous work from different teams, our results suggest a consistent model for the activation of Sub1 in several steps (Fig. 7). Initially, the inactive Sub1 precursor undergoes a first auto-catalytic cleavage at Asp217, between its PD and catalytic domain. This primary maturation takes place within the ER (41) and is required for protein dimerization, because an uncleaved propeptide within the active site would sterically clash with the position of the belt subunit in the dimer (see Fig. 5a). Consistently, a mutant form of Sub1 unable to cleave itself at this primary maturation site—and therefore unable to dimerize—was found to accumulate in the ER (45), strongly suggesting that Sub1 dimerization is essential for exoneme transport. This is fully consistent with the observation that Sub1 was mainly found as a dimer in *P. falciparum* mature schizonts (Fig. 6).

It has been recently reported that the aspartic protease PmX cleaves the Sub1 PD, as an intact form of PD (p31) could be detected in parasite lysates lacking PmX (45). In agreement with the acidic environment of secretory vesicles (55, 56), the exoneme-localized PmX requires acidic conditions for activity (43, 44, 57, 58), implying that Sub1 PD cleavage should occur during exoneme transport, before its discharge into the rather neutral PV (59), which is the site of Sub1 action (7, 60). In other words, PD processing and Sub1 activation appear to occur at different times in different cell compartments. The results reported here strongly suggest that this spatiotemporal dissociation is made possible by pH-dependent Sub1 dimerization. According to this model (Fig. 7), the dimeric assembly of Sub1 is tightly maintained within the acidic environment of the exoneme, even after PmX processing, to preserve not only the structural integrity of the protease but also its complete inactivation during transport. This hypothesis is consistent with the crystal structures, as the two PmX cleavage sites detected in the PD, respectively, in the middle of the N-terminal helix of the belt subunit and in a solvent-exposed loop from the bacterial PD-like subunit (45) are on the surface of the protein and are not expected to interfere with dimer formation.

Upon the cGMP-mediated drastic shift in [Ca^2^] that immediately precedes the egress of merozoites (4), Sub1 is discharged into the PV (6). Once there, the increase of pH would strongly destabilize Sub1 dimers and promote the emergence of a monomeric Sub1 fraction and the subsequent clearance of PD fragments (45), leading to full activation. Active Sub1 may then contribute to further auto-catalytic processing of both the PD, as two cleavage sites have been detected in the PvS1 belt subunit in the presence of calcium (47), and the catalytic domain, as the conversion of the p54 into the p47 species at Asp249 (41) can also take place in both compartments, either by PmX in the exoneme or by auto-catalysis in the PV (45). Essentially, this N-terminal processing of the catalytic domain corresponds to cleave off the poorly conserved (47) unstructured region from the core subtilase domain (Fig. 7) and was recently shown to be dispensable for parasite growth (45).

For the subtilase family in general, the displacement of PDs following primary automaturation is possibly the most important and rate-limiting single event for activation. These may involve variations of [Ca^2^] and/or pH, as described for bacterial subtilisins (23) or for eukaryotic furin and PC1/3, which undergo subtle pH-induced conformational changes that regulate their activation within secretory vesicles (27, 28). Variations in pH also participate in removing the PD of plant subtilisins (29) and a lower pH during secretion participates in activating yeast proteinase A and procarboxypeptidase Y (61, 62). For Sub1, *Plasmodium* appears to use a novel pH-dependent two-step strategy, as activation requires both an endogenous activating protease, PmX, to cleave the PD and a pH increase (arising from the discharge of Sub1 into a different cell compartment) to relieve inhibition. Although it is unclear why the malaria parasite does it this way, this novel regulatory element of subtilisin activation — so far only observed in *Plasmodium* — is part of a more complex process (involving several enzymes, cellular compartments, and signaling events) required to ensure the egress of competent merozoites from RBCs and allow *Plasmodium* propagation in the host blood and subsequent vector-borne transmission.

## MATERIALS AND METHODS

### Production and purification of the PfS1_FL_, PvS1_FL_, PvS1_CD_, and PvS1_ΔBelt_ recombinant enzymes

The baculovirus-expressed recombinant PfS1_FL_, PvS1_FL_, and PvS1_ΔBelt_ (Genbank accession codes FJ536585, JX491486, and KM211548, respectively) were prepared as described (47, 63, 64). Briefly, culture supernatant was harvested 3 days post-infection with recombinant baculoviruses, centrifuged 30 min at 2,150 × *g* and concentrated/diafiltrated against loading buffer (D-PBS, 500 mM NaCl, 5 mM Imidazole) using the AKTA crossflow system (GE Healthcare) supplemented with a 10 KDa, 1.2ft2 Kvicklab cassette (GE Healthcare).

The proteins were purified on an AKTA purifier system (GE Healthcare) at 4°C. The sample was loaded onto a 5-mL TALON metal affinity resin (Clontech) equilibrated in loading buffer. After extensive washes with loading buffer, the bound protein was eluted with a linear gradient of 5–200 mM imidazole in D-PBS, 500 mM NaCl. Purified protein-containing fractions were pooled and concentrated by using Amicon Ultra 15 (molecular weight cutoff of 10,000) and size-fractionated onto a HiLoad 16/60 Superdex 75 column equilibrated with 20 mM Tris, 500 mM NaCl, 1 mM CaCl_2_ (pH 8). Fractions were monitored by absorbance (280 nm) and analyzed by Coomassie Blue staining of SDS-polyacrylamide gels and enzyme activity assay. For enzymatic assays, purified recombinant proteins PvS1_FL_ and PvS1 catalytic domain (PvS1_CD_), prepared as described (65), were stored at −20°C following the addition of 10% vol/vol of pure glycerol. Protein concentrations were determined from *A*_280_, using the extinction coefficient predicted by ExPASy ProtParam (66).

### Protein crystallization

Initial identification of crystallization conditions was carried out using the vapor diffusion method in a Cartesian technology workstation. Sitting drops were set using 200 nL of 1:1 mixture of PfS1_FL_ and a crystallization solution (672 different conditions, commercially available), equilibrating against 150 µL reservoir in a Greiner plate. Optimization of initial hits was pursued manually in Linbro plates with a hanging drop setup. The best crystals were obtained by mixing 1.5 µL of native PfS1_FL_ (9.7 mg/mL) with 1.5 µL of the reservoir solution containing 1.6 M NaH_2_PO_4_/0.4 M K_2_HPO_4_ and 0.1 M phosphate-citrate, pH 5.2, at 18°C. Rod-like crystals appeared within 3–4 weeks and had dimensions of 0.25 × 0.05 × 0.05 mm^3^. Prior to diffraction data collection, single crystals were flash-frozen in liquid nitrogen using a mixture of 50% paratone and 50% paraffin oil as cryoprotectant.

### Data collection, structure determination, and refinement

X-ray diffraction data were collected at 100K using beamline Proxima1 (wavelength = 1.07169 Å) at the SOLEIL synchrotron (France). All data sets were processed using XDS (67) and AIMLESS from the CCP4 suite (68). The crystal structure was determined by molecular replacement methods using Phaser (69) and *P. vivax* Sub1 (PDB code 4tr2) as the probe model. Crystallographic refinement was done through iterative cycles of manual model building with COOT (70, 71) and reciprocal space refinement with BUSTER (72) using a TLS model and non-crystallographic symmetry restraints. The crystallographic statistics are shown in Table 1. Structural figures were generated with Chimera (73) or Pymol (The PyMOL Molecular Graphics System, Version 2.0 Schrödinger, LLC). All surface areas calculation was done with program PISA (Protein Interfaces, Surfaces and Assemblies) (74). Atomic coordinates and structure factors of PfS1_FL_ have been deposited in the protein data bank under the accession code 8POL.

### Analytical ultracentrifugation

Prior to AUC analysis, protein samples were dialyzed against buffer pH 8 (50 mM Tris pH 8, 150 mM NaCl, and 10 mM CaCl_2_) or buffer pH 5 (50 mM NaCit pH 5, 150 mM NaCl, 10 mM CaCl_2_) at 4°C for 24 h. Protein samples were centrifuged in a Beckman Coulter XL-I analytical ultracentrifuge at 20°C in a four-hole rotor AN60-Ti equipped with 12 mm six sectors centerpieces. Detection of the protein concentration as a function of radial position and time was performed by optical density measurements at 280 nm. For sedimentation equilibrium experiments, 120 µL of PvS1_FL_ or PvS1_ΔBelt_ (2, 5, and 10 µM) in buffer pH 8 or buffer pH 5 were spun sequentially at rotor speeds of 9,000, 13,000, and 16,000 rpm. The data were acquired after reaching equilibrium for 1 h at each speed. The following parameters were calculated using Sednterp 1.09 and used for the analysis of the experiment: partial specific volume of 0.732 mL g^−1^, viscosity *η* = 1.068 cP, and density *ρ* = 1.0139 g mL^−1^.

Sedimentation equilibrium radial distributions were analyzed by global fitting using the single-species model with Ultrascan 9.5 software (http://www.ultrascan.uthscsa.edu) (75). Monte Carlo analysis was performed on the fit in order to estimate measurement error.

### PvS1 enzymatic activity

The activities of PvS1_FL_, PvS1_ΔBelt_, and PvS1_CD_ were assessed as previously described (63), at 37°C for 60 min in 10 mM CaCl_2_, 150 mM NaCl at different pH. Briefly, the reaction was initiated by adding 20 µM of a FRET substrate (Dabsyl-KLVGADDVSLA-EDANS) and followed by measuring the EDANS fluorescence (excitation at 360 nm and emission at 500 nm) every 2 min under shaking over 1 h using a Tecan Infinite M1000 spectrofluorimeter. All measurements were performed in triplicate.

### Culture of *P. falciparum* parasites, western blots, and immunofluorescence analysis

Parasites were cultured as previously described (63) in RPMI 1640 medium containing L-glutamine, 25 mM HEPES (Invitrogen) supplemented with 0.5% Albumax II (Gibco), with a hematocrit of human red blood cells of 5%, 100 µM hypoxanthine (C.C.pro, Germany), 25 µg/mL gentamycin (Sigma-Aldrich) at 37°C in a 5% O_2_, 5% CO_2_, and 90% N_2_ atmosphere. To obtain human red blood cells, human peripheral blood samples were collected from healthy volunteers through the ICAReB platform (Clinical Investigation & Access to Research Bioresources) from the Center for Translational Science, Institute Pasteur (76). All participants received an oral and written information about the research and gave written informed consent in the frame of the healthy volunteers Diagmicoll cohort (Clinical trials NCT 03912246) and CoSImmGEn cohort (Clinical trials NCT 03925272), after approval of the Ethics Committee of Ile-de-France (30 April 2009 and 18 Jan 2011, respectively).

*P. falciparum* schizonts, enriched for mainly mature forms prior to merozoite egress, were obtained as previously described (77) from a parasite culture on Percoll 75% prior to be incubated in standard culture conditions until the expected parasite stages are obtained. Following washes of the mature segmented schizonts in PBS, proteins were extracted in n-dodecyl-β-D-maltoside (DDM, Merck) 1% detergent, diluted in PBS supplemented with protease inhibitors (Complete, Roche) for 30 min on ice and centrifuged at 16,100 × *g* at 4°C for 15 min. After addition of native sample buffer (0.1% Ponceau S/50% glycerol), the protein extracts were separated on a NativePAGE 4-16% Bis-Tris using NativePAGE 20X Running Buffer (Invitrogen) for the anode buffer, supplemented with DOC (Na-Deoxycholate, Sigma) 0.05% and 0.01% DDM for the cathode buffer. After migration (80V/1 h plus 150V/2h30), polyacrylamide gels were incubated 15 min at room temperature with agitation in a denaturation buffer composed of transfer buffer (Bio-Rad) supplemented with 2% SDS and 10 mM DTT (Sigma). Proteins were then transferred to a nitrocellulose membrane using a Trans-Blot Turbo (Bio-Rad). The NativeMark Unstained Protein Standard (Invitrogen) is revealed by incubation of the membrane in a 0.2% Ponceau S solution (Serva). For SDS page conditions, the same samples were separated and transferred as previously described (63).

The full-length PfS1 recombinant purified protein was used to immunize rabbits subcutaneously following a 2-month standard procedure (Proteogenix, France). Briefly, the first immunization by the purified protein emulsified with the complete Freund’s adjuvant was followed by two boosts with incomplete Freund’s adjuvant at days 39 and 53 and the serum was collected at day 70 post the initial injection and kept in 50% glycerol at −20°C.

Blots were incubated with antibodies (1/3,000 dilution) purified on G-protein (Proteogenix, France), followed by horseradish peroxidase-conjugated (HRP) secondary rabbit antibodies (Promega, 1/25,000) and revealed by chemiluminescence (Pierce) using ChemiDoc and ImageLab (BioRad).

Immunofluorescence analysis was performed as previously described (63) using Percoll-purified *P. falciparum* segmented schizonts washed in PBS, deposited on glass slides and air-dried before to be fixed with paraformaldehyde 4% and permeabilized with 0.1% Triton X100 (Sigma). Slides were saturated with 1% Albumax II overnight at 4°C before successive incubations with the purified anti-PfS1 and the anti-MSP1 mouse mAb 27G2 both diluted to 1:1,000. Secondary antibodies Alexa Fluor 488-conjugated anti-rabbit IgG and Alexa Fluor 594-conjugated anti-mouse IgG (Invitrogen) were used diluted to 1:1,000. Antibodies were prepared in PBS containing Albumax II 1%. Hoescht 33342 (Invitrogen) diluted at 1:5,000 was added to the secondary antibody solutions. PBS-washed slides were sealed with Vectashield (Vector laboratories). Images were collected using a Leica DM5000B microscope with an 1,000× magnification.

## SUPPLEMENTAL MATERIAL

Supplemental figures mbio.00198-24-s0001.pdf Figures S1 to S3.

PDB Report mbio.00198-24-s0002.pdf PDB report of data describing the 3D structure of *Plasmodium falciparum* Sub1 protease.

ASM does not own the copyrights to Supplemental Material that may be linked to, or accessed through, an article. The authors have granted ASM a non-exclusive, world-wide license to publish the Supplemental Material files. Please contact the corresponding author directly for reuse.
