# Supplementary material for: Prodomain-driven enzyme dimerization: a pH-dependent autoinhibition mechanism that controls Plasmodium Sub1 activity before merozoite egress
Source: mBio. 2024 Feb 22;15(3):e00198-24. doi: 10.1128/mbio.00198-24 (PMC10936178; doi:10.1128/mbio.00198-24)
Supplement: Supplemental figures — Figures S1 to S3. [file mbio.00198-24-s0001.pdf]

Prodomain-driven enzyme dimerization: a pH-dependent autoinhibition mechanism that controls *Plasmodium* Sub1 activity before merozoite egress

Mariano Martinez[*1], Anthony Bouillon[*1], Sébastien Brule[2], Bertrand Raynal[2], Ahmed Haouz[3], Pedro M. Alzari[1#] & Jean-Christophe Barale[1#]

SUPPLEMENTAL INFORMATION

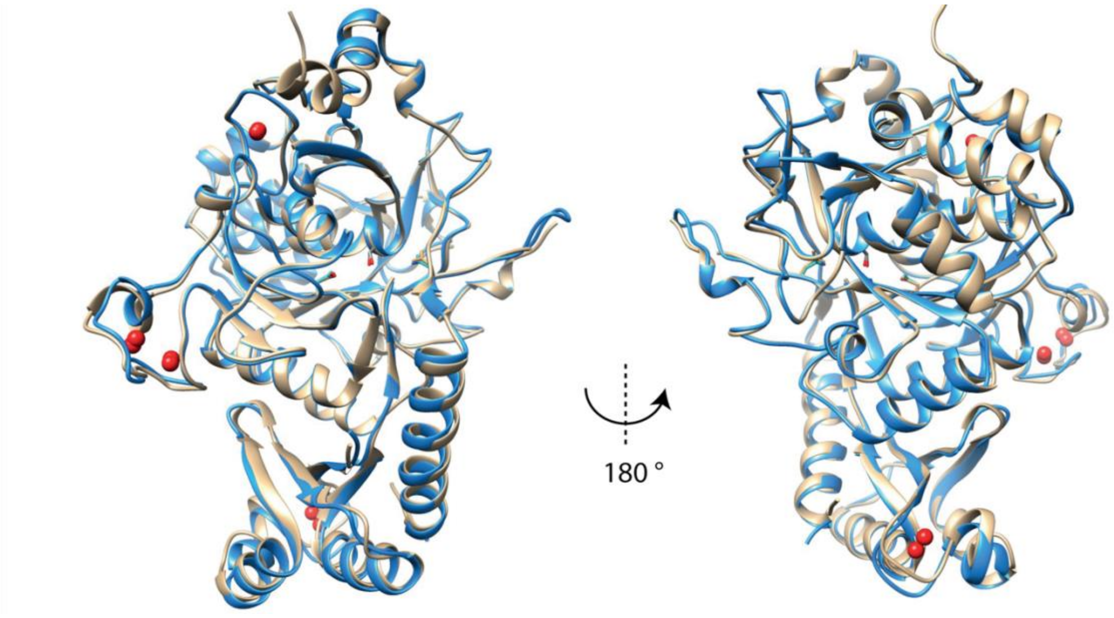

Figure S1. Structural superposition of the PfS1$_{FL}$ (brown, this work) and PvS1$_{FL}$ (blue, PDB code 4tr2) monomers, which share 71% of amino acid sequence identity.

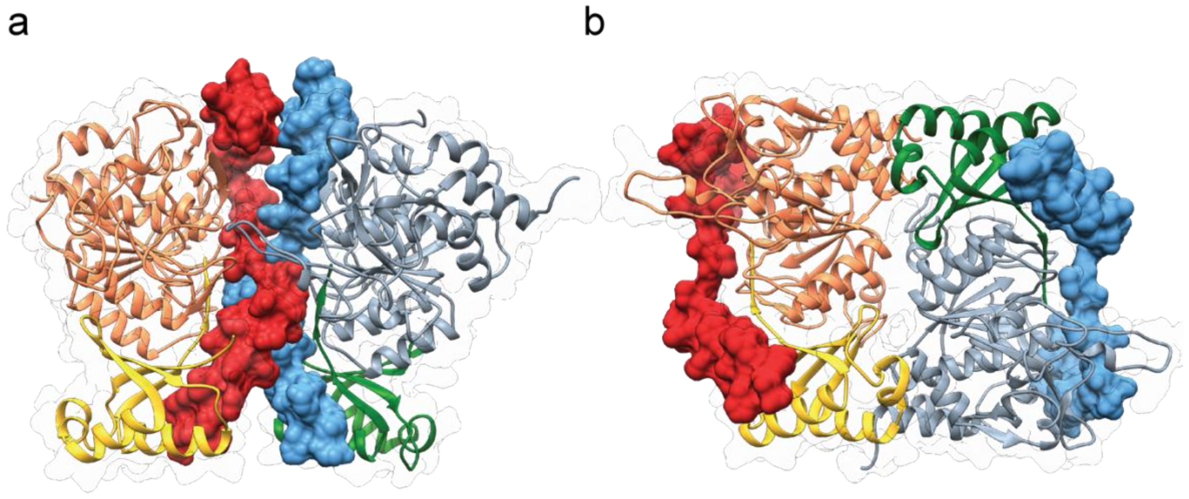

Figure S2. Two possible homodimeric arrangements suggested by lattice contacts in tetragonal PvS1$_{FL}$ crystals (PDB code 4tr2, (47). **(a)** Putative dimeric arrangement with the active site clefts face-to-face and the belt subunits (shown in molecular surface representation) at the center of the interface. **(b)** Putative dimeric arrangement with the active site clefts pointing away from each other (back-to-back conformation). In this arrangement, the belt subunits are not involved in intermolecular contacts. In both cases, the PD and the catalytic domain are colored pink and red in one protomer, and cyan and blue in the other protomer.

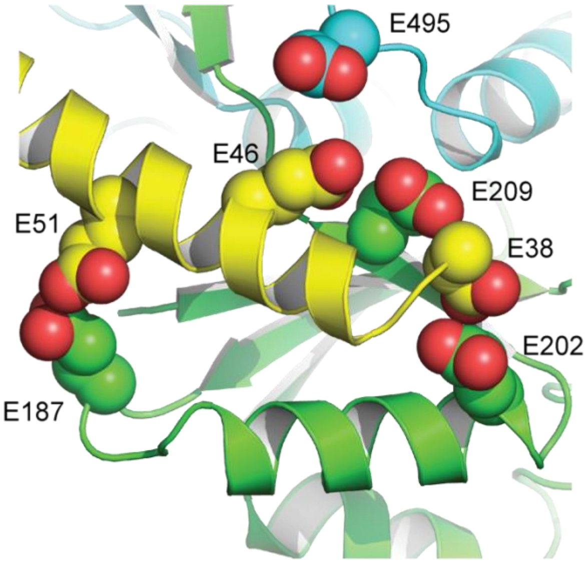

Figure S3. Close view of the interaction between the N-terminal belt helix (in yellow) with the bacterial-like prodomain (green) and the catalytic domain (cyan) of PfS1$_{FL}$. Seven glutamic acids (shown in sphere representation and labelled) have their carboxylate groups in close proximity to each other. Five of these (E46, E187, E202, E209 and E495) are predicted to be protonated at pH 5, likely aiding the interaction observed in the crystal structure.
